# Apelin/APJ system: A key therapeutic target for liver disease

**DOI:** 10.18632/oncotarget.22841

**Published:** 2017-12-01

**Authors:** Shuang-Yu Lv, Binbin Cui, Wei-Dong Chen, Yan-Dong Wang

**Affiliations:** ^1^ Key Laboratory of Receptors-Mediated Gene Regulation and Drug Discovery, School of Medicine, Henan University, Kaifeng, Henan, P. R. China; ^2^ State Key Laboratory of Chemical Resource Engineering, College of Life Science and Technology, Beijing University of Chemical Technology, Beijing, P. R. China; ^3^ Key Laboratory of Molecular Pathology, School of Basic Medical Science, Inner Mongolia Medical University, Hohhot, Inner Mongolia, P. R. China

**Keywords:** apelin, APJ, liver, cirrhosis, fibrosis

## Abstract

Apelin, a new bioactive peptide, was identified as an endogenous ligand for APJ (Angiotensin II receptor-like 1). Apelin and its receptor have an abundant distribution in central nervous system and peripheral tissues, including liver. Apelin/APJ has diverse physiological and pathological effects, including regulation of cardiovascular function, angiogenesis, fluid homeostasis and so on. Apelin/APJ system may act as a novel potential therapeutic target for liver disease. In this article, we review the role of apelin/APJ system in liver fibrosis, hepatitis, hepatic cirrhosis, liver injury and metabolic liver disease.

## INTRODUCTION

APJ (Angiotensin II receptor-like 1) was discovered by O’Dowd in 1993 [1]. The gene encoding APJ is intronless in the coding region and its approved gene symbol is *APLNR* in human and *Aplnr* in the mouse and the rat [2]. In human, *APLNR*, located on chromosome 11q12, encodes a 380-amino acid protein [1, 2]. In the mouse and the rat, *Aplnr*, present at the chromosomes 2E1 and 3q24, respectively, encodes 377-amino acid proteins [3-5]. The human APJ is a G-protein-coupled receptor (GPCR) and the similarities of primary structure of APJ and angiotensin II receptor type 1 (AT1R) is 31%. However, angiotensin II could not combine with APJ [1].

Apelin, firstly extracted from bovine stomach, was confirmed as an endogenous ligand for APJ [6]. The human apelin gene expresses a 77-amino acid prepropeptide, called preproapelin, and is located on chromosome Xq25-26 [6]. The preproapelin contains a signal peptide at its N-terminal, directing the secretory pathway of apelin. The preproapelin, including several binding sites for endopeptidases to cut out, was processed and generated to different active fragments, such as apelin (66–77), named apelin-12, apelin (65–77), named apelin-13, apelin (61–77), named apelin-17, and apelin (42–77), named apelin-36 [4, 7]. [pGlu]apelin-13, the pyroglutamylated form of apelin-13, has been reported to be protected from exopeptidase degradation [4]. Among the different forms of apelin fragments, apelin-13 and [pGlu]apelin-13 are the most potent activators for apelin receptor expressed in cell lines [6-9].

Apelin and APJ are highly expressed in the central nervous system (CNS) and peripheral tissues, such as adipose tissue, brain, liver, lung, kidney, and the cardiovascular system in both human and rodents [10]. Apelin and APJ are also expressed in adult rat liver [5]. In rat liver, the positive staining of apelin has been detected in the endothelia of the portal and central veins, and the Kupffer cells using immunohistochemical staining [11]. Recently, Principe et al. showed that apelin and APJ have an abundant distribution in hepatic stellate cells (HSCs) and hepatocytes of the rat cirrhotic liver [12]. Furthermore, APJ is also highly expressed in hepatocytes of human cirrhotic liver [13]. Hence, the apelin/APJ system may produce important effects on physiology and pathophysiology of liver function [13].

Apelin/APJ has diverse physiological and pathological effects, including regulation of cancer [14], vascular smooth muscle cells (VSMC) proliferation [15], ischemia/reperfusion injury [16], fluid homeostasis [17], cardiovascular function, angiogenesis, and acting as a neuroendocrine modulator of the hypothalamic-pituitary-adrenal (HPA) axis in response to stress in human or animals [2]. Apelin/APJ signaling is important for embryonic angiogenesis and is up-regulated during tumor angiogenesis [18]. Apelin promotes proliferation and migration of vascular endothelial cells, and could stimulate vascular sprouting *in vivo* even in the absence of vascular endothelial growth factor [19].

In the current work, we have reveiwed the latest research progress about the role of apelin/APJ system in liver disease, including liver fibrosis, hepatitis, hepatic cirrhosis, liver injury, metabolic liver disease and fatty liver disease.

## APELIN IN LIVER FIBROSIS AND HEPATITIS

The major feature of patients with chronic liver disease is hepatic architectural disruption. Actually, the liver of these patients undergoes an intense process of tissue remodelling characterized by chronic inflammation, neoangiogenesis and fibrogenesis [20]. In LX-2 cells, a cell line derived from human HSCs, proinflammatory substances induced the expression of apelin gene [21]. In HSCs, the overexpression of APJ was activated by platelet-derived growth factor (PDGF) and proinflammatory cytokines, suggesting that APJ might promote vascular remodeling in fibrogenesis [22].

**Figure 1:**
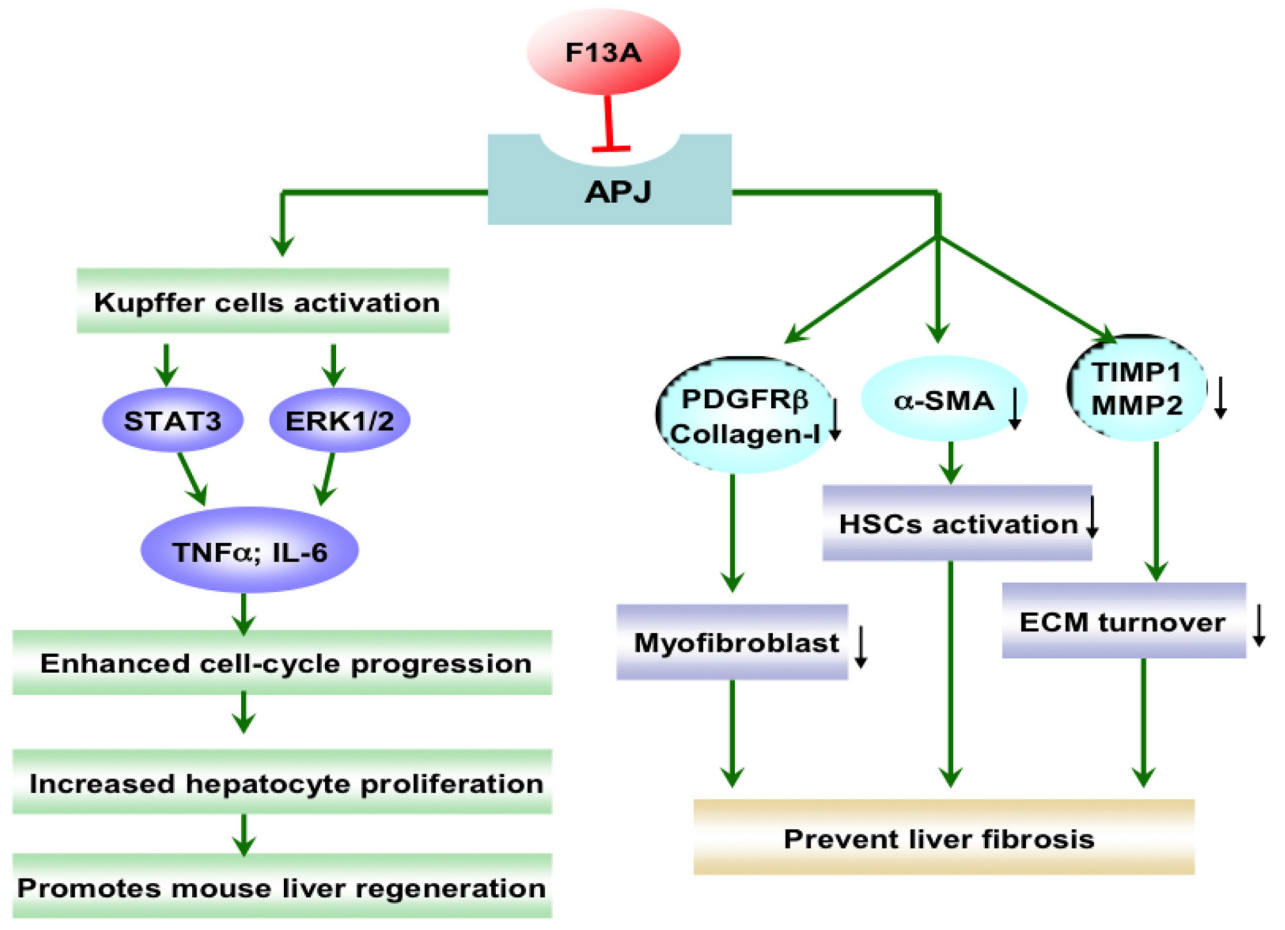
The effect of apelin/APJ system blockade on liver fibrosis and liver regeneration F13A, apelin-13(F13A); PDGFRβ, platelet-derived growth factor receptor β; ɑ-SMA, ɑ-smooth muscle actin; TIMP, tissue inhibitor of matrix metalloproteinase; MMP, matrix metalloproteinase; HSCs, hepatic stellate cells; ECM, extracellular matrix; STAT3, signal transducer and activator of transcription 3; ERK, extracellular signal-related kinase; TNF, tumor necrosis factor; IL-6, interleukin-6; ↓, decrease. Green arrow denotes stimulation. Red arrow denotes suppression.

In LX-2 cells, both profibrogenic molecules angiotensin II and endothelin-1 enhanced apelin expression, and apelin could increase the synthesis of collagen-I and platelet-derived growth factor receptor (PDGFR). Moreover, APJ receptor antagonist F13A drastically reduced collagen-I and PDGFR expression stimulated by angiotensin II and endothelin-1 [21]. Apelin was involved in regulating fibrogenic activity induced by angiotensin II and endothelin-1, and apelin would be an essential regulator of fibrogenesis in human liver disease [21]. The roles of apelin/APJ system in liver fibrosis were shown in Figure 1. In CCl_4_-treated rats as a fibrosis animal model, F13A reduced hepatic collagen content, improved mean arterial pressure (MAP) and portal pressure (PP), ameliorated cell viability, and inhibited angiogenesis and cell infiltrate [23]. These effects were associated with reduction of PDGFRβ, ɑ-smooth muscle actin (ɑ-SMA), matrix metalloproteinases, and tissue inhibitors of matrix metalloproteinase [23]. Chen et al. has reported that there is a significant linear correlation between the apelin mRNA level and liver fibrosis, serum total bilirubin and the grade of esophageal varices. The hepatic apelin/APJ system is activated in the progression of biliary atresia (BA), particularly in end-stage cirrhosis [24]. The expression level of apelin indicates the degree of hepatic fibrosis and esophageal varices, so it could be potentially considered as a prognostic factor for BA patients [24]. Considering the close relationship between apelin expression and profibrogenic factors in HSCs, and the alleviation of fibrogenesis and angiogenesis under the condition of APJ blockade in hepatic fibrosis, the apelin/APJ system might be a promising therapeutic target for liver fibrosis.

Apelin level varies at the different stages of chronic hepatitis C (CHC), which may lead to fibrosis progression. It was found that TNF-α was negatively correlated to adjusted apelin in CHC patients [25]. Apelin is expressed in the liver of hepatitis-C virus (HCV) patients but not healthy individuals and it is involved in the disease progression [26].

## APELIN AND HEPATIC CIRRHOSIS

The circulating level of apelin was increased in patients with cirrhosis compared with healthy subjects [12]. In addition, apelin protein and gene were upregulated in cirrhotic liver compared with normal liver in humans [27]. Recent clinical study demonstrates that the serum apelin level showed a significant relationship with the severity of liver cirrhosis in patients with chronic liver disease (CLD) [28]. In rats with cirrhosis, the apelin levels were higher than controls, and apelin as well as APJ mRNA also showed an obvious rise in hepatic tissue [12]. Treatment with the F13A, an apelin receptor antagonist, alleviated hepatic fibrosis and vessel density and improved cardiovascular performance in rats with cirrhosis. These results suggest that blocking apelin/APJ signaling pathway might contribute to treatment of cirrhosis and related diseases [12]. In the sinusoid lining cells of human cirrhotic liver, the APJ mRNA and protein expression was upregulated. The highly expressed APJ was detected in activated HSCs, myofibroblasts, and fibroblasts in Child-C cirrhosis liver [22]. These results indicate that APJ might have effect on vascular remodeling and increased portal hypertension in cirrhosis [22]. The apelin/APJ was activated in patients with cirrhosis and blocking apelin/APJ system could alleviate symptoms of hepatic cirrhosis, suggesting that apelin/APJ system is a potential therapeutic target of hepatic cirrhosis.

In cirrhosis, proliferation of hepatic arterial capillaries leads to an acceleration of arterial blood pouring into the sinusoids, increasing the sinusoidal blood pressure in relation to the defenestration of sinusoidal endothelial cell and consequently leading to the portal hypertension in liver cirrhosis [27, 29]. Apelin was weakly expressed in hepatic sinusoidal endothelial cells and in proliferated arterial capillaries directly connecting to the sinusoids in early stage cirrhotic liver, while apelin was strongly expressed in proliferated arterial capillaries in end stage cirrhosis [27]. Additionally, apelin levels were significantly correlated with the severity of liver disease in patients with alcoholic cirrhosis. Apelin, diponectin and RBP4 levels are deregulated in liver cirrhosis depending on the degree of liver dysfunction [30].

The progression of liver fibrogenesis and cirrhosis is dependent on the different dynamic inflammatory state [31]. With extension and distortion of the normal hepatic architecture of liver fibrosis, tissue hypoxia becomes an essential regulator of the production of proinflammatory and proangiogenic factors [32]. Hypoxia and proinflammatory factors induce the expression of APJ in LX-2 and HepG2 cells. APJ activation stimulates the expression of angiopoietin-1 in LX-2 cells, which triggers the generation of vascular endothelial growth factor type A and PDGF BB in HepG2 cells. In addition, activation of APJ promotes the HSCs proliferation. The authors proposed that the activation of the apelin system induced by hypoxia and inflammatory factors contributes to angiogenic and fibroproliferative response in chronic liver disease [13].

In addition, apelin and APJ are overexpressed in hepatocellular carcinoma. The apelin/APJ induces tumor arteriogenesis, and they could work as a signal for arteriogenesis in hepatocellular carcinoma [33]. In hepatoma HepG2 cells, apelin promotes autophagy by inducing the phosphorylation of ERK1/2 and upregulating the expression of Beclin1 [34].

## APELIN/APJ IN LIVER INJURY AND LIVER REGENERATION

Yasuzaki et al. found that mRNA levels of apelin and APJ in the liver were increased in mice with fas-mediated liver injury [35]. In APJ^-/-^ mice, the liver injury and apoptotic changes were markedly reduced and the obvious activation of JNK was completely abolished after treatment of Jo2, a fas-agonistic antibody, comparing with WT mice, indicating that apelin/APJ signaling may facilitate fas-induced liver injury at least partially via JNK activation [35]. As we know, the JNK activity is also required for the development of liver cancer in carcinogenesis [36]. In hepatic ischemia reperfusion (I/R) injury rats, apelin-13 as well as leptin produced protective effects. Leptin and apelin treatments reduced lipid peroxidation, alleviated histological tissue damage and improved liver function for hepatic I/R injury rats [37]. However, its underlying mechanism remains to be elucidated.

Liver regeneration is a process of adaptive growth caused by specific stimuli and involves replication of the liver cells, mainly hepatocytes [38]. It is reported that abolishment of apelin/APJ system promotes mouse liver regeneration. Blockade of the apelin/APJ system using F13A promoted hepatocyte proliferation, increasing the secretion of TNF-ɑ and IL-6 by promoting the activation of Kupffer cells during early phase of liver regeneration, showing therapeutic effects in a mouse extended partial hepatectomy (ExPH) model [39] (Figure 1). The apelin/APJ system has an abundant distribution in liver and APJ knock out or abolishment could promote liver regeneration and relieve liver injury. Consequently, the apelin/APJ system would potentially be as a target for the treatment of liver injury and liver regeneration.

## APELIN/APJ IN METABOLIC LIVER DISEASES

**Figure 2:**
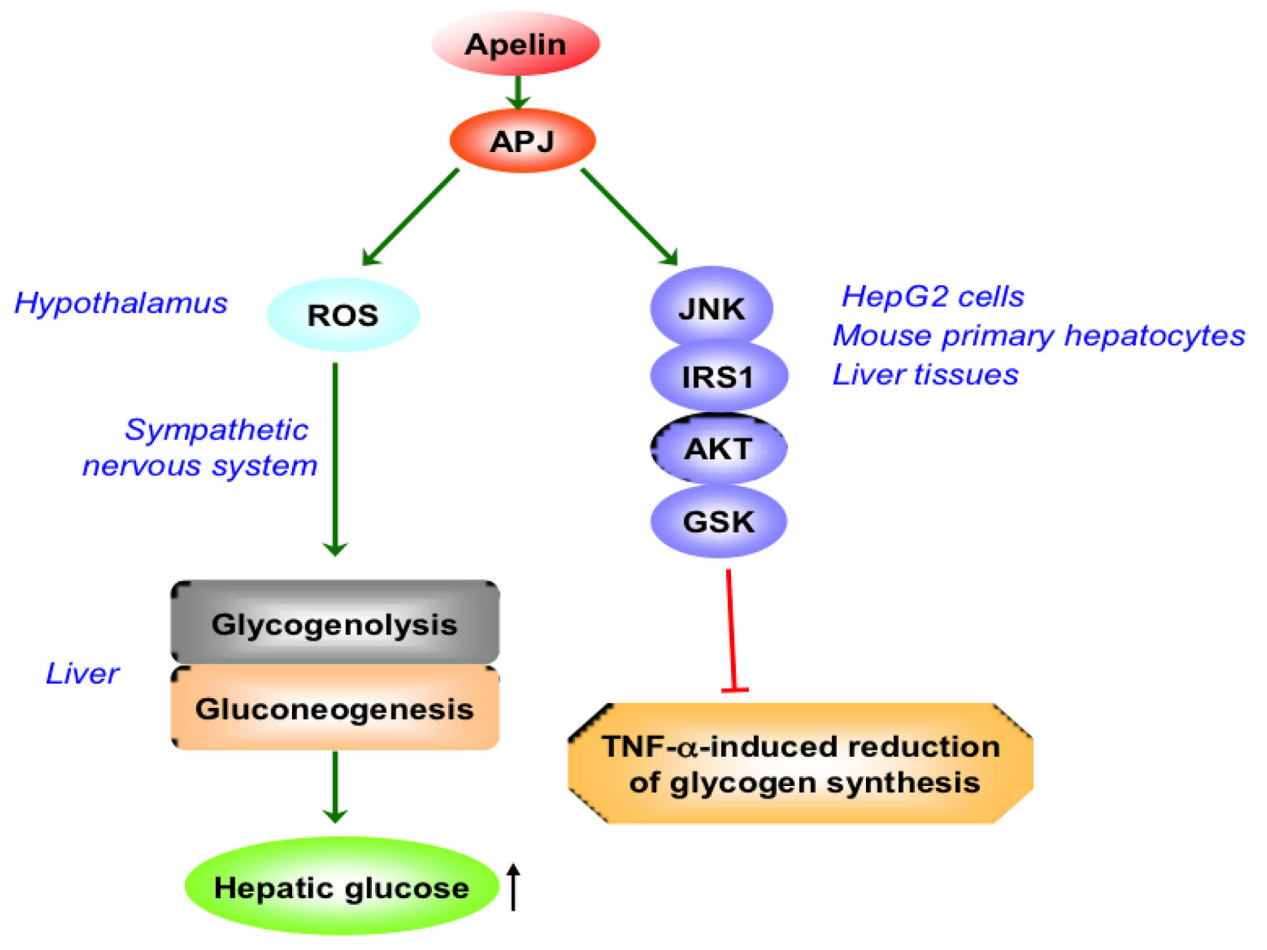
The role of apelin/APJ system on hepatic glucose metabolism ROS, reactive oxygen species; JNK, c-Jun N-terminal kinase; IRS-1, insulin receptor substrate-1; GSK, glycogen synthase kinase; TNF, tumor necrosis factor;↑, increase. Green arrow denotes stimulation. Red arrow denotes suppression.

Butruille et al. found that the apelin/APJ system is changed mostly in adipose tissue, liver and kidney in obese and insulin-resistant female mice, suggesting that apelin/APJ system may be involved in regulating pathologic status of these tissues under the condition of obesity and diabetes [40]. APJ was detected in HepG2 cells, mouse primary hepatocytes and mouse liver tissue. Apelin reversed the decrease of glycogen level induced by TNF-ɑ, which was mediated by JNK-IRS1-AKT-GSK pathway in HepG2 cells, hepatocytes and liver tissue of mice [41]. This effect was blocked by F13A, suggesting that APJ was involved in the improvement of apelin on the reduction of glycogen synthesis induced by TNF-ɑ [41]. However, hypothalamic apelin could lead to deleterious effect for liver with diabetes. I.c.v. injection with apelin stimulates liver glycogenolysis and gluconeogenesis via the sympathetic nervous system (SNS), inducing peripheral hyperglycemia in normal mice. And the regulation of central apelin on glycemia was dependent on the APJ receptor and the production of hypothalamic reactive oxygen species (ROS) [42] (Figure 2).

Apelin, recognized as an adipokine, was shown to be correlated with nonalcoholic fatty liver disease (NAFLD). Ercin et al. showed that plasma levels of apelin-12 were higher in patients with NAFLD than in healthy comparison subjects [43]. Moreover, the apelin level has a positive correlation with BMI and homeostasis model assessment (HOMA) indexes in subjects with NAFLD [43]. Additionally, the serum levels of apelin-36 were markedly higher in NAFLD patients than in healthy individuals [44]. In NAFLD patients, serum apelin-36 levels showed a slight relation with HOMA of insulin resistance [44]. Liver malondialdehyde (MDA) level was significantly correlated with subcutaneous apelin gene expression in rats with high-fat feeding [45].

## CONCLUSIONS AND PERSPECTIVES

In summary, activation of APJ receptor seems to promote development of liver disease, whereas blocking APJ receptor signaling could be used for treatment of liver diseases, such as hepatic cirrhosis and liver injury, and it was beneficial to liver regeneration. Further studies on the mechanism underlying the effect of apelin-APJ signalling in liver disease need to be investigated. Although the experimental evidence supporting the roles for apelin/APJ in liver diseases is overwhelming, the clinical evidence is limited. Further studies were wanted to determine whether these agents could be used in humans undergoing liver disease or surgery. In addition, development of the more effective antagonists and the more stable nonpeptidic APJ antagonists might provide us a new therapeutic tool for liver disease in the future.
